# Plasma Granzyme B in ST Elevation Myocardial Infarction versus Non-ST Elevation Acute Coronary Syndrome: Comparisons with IL-18 and Fractalkine

**DOI:** 10.1155/2013/343268

**Published:** 2013-11-06

**Authors:** Hala O. El-Mesallamy, Nadia M. Hamdy, Adel K. El-Etriby, Eman F. Wasfey

**Affiliations:** ^1^Biochemistry Department, Faculty of Pharmacy, Ain Shams University, Abassia, Cairo 11566, Egypt; ^2^Cardiology Department, Faculty of Medicine, Ain Shams University, Abassia, Cairo 11566, Egypt

## Abstract

*Objective*. The proapoptotic protein, granzyme B (GZB), was identified as a contributor to the atherosclerotic plaque instability and recently as inflammatory activator. We studied the release kinetics of GZB and other markers of inflammation such as high sensitivity C reactive protein (hsCRP), interleukin 18 (IL-18), and fractalkine (FKN) in the early phase after acute cardiac events in different ACS subgroups. *Methods*. Thirty-six nondiabetic patients with ACS were compared to 12 control subjects. According to ACS diagnosis, the patients were classified into 22 patients with ST elevation myocardial infarction (STEMI) and 14 patients with non-ST elevation myocardial infarction or unstable angina (NSTEMI/UA). Blood samples were taken on day 1 (day of onset) and day 3 to measure hsCRP, IL-18, FKN, and GZB by ELISA. *Results*. Patients with ACS showed significantly higher GZB, IL-18, and FKN levels than the controls. STEMI group showed significantly higher GZB levels than NSTEMI/UA group. On day 3, FKN levels displayed a significant decrease, while GZB levels were significantly increased. IL-18 levels were more or less constant. GZB levels were positively correlated with IL-18 (*r* = 0.416, *P* < 0.01) and FKN (*r* = 0.58, *P* < 0.001). *Conclusions*. Unlike IL-18 and FKN, plasma GZB may be a marker of ACS disease severity.

## 1. Introduction

Acute coronary syndrome (ACS) remains a major cause of mortality and morbidity [1]. It is characterized by acute inflammatory response which causes coronary plaque rupture with subsequent thrombosis [2]. Accumulating evidence showed that inflammation and immune cell activation play a key role in collagen loss in the fibrous cap which is a prelude to fibrous cap rupture [3, 4]. The extent of cardiac damage due to this inflammatory response is reflected by peripheral levels of inflammatory biomarkers involved in the process of plaque instability [5]. Moreover, markers of plaque destabilization and plaque rupture such as high sensitivity C-reactive protein (hsCRP) may be used to predict future cardiovascular events not only in apparently healthy subjects, but also in patients with ACS [6]. 

Granzyme B (GZB) is a serine protease released from cytotoxic T lymphocytes (CTLs) and natural killer (NK) cells, playing an important role in cellular apoptosis by activating intracellular caspases [7]. Furthermore, GZB is also identified as an extracellular protease degrading specific extracellular substrates such as fibronectin, vitronectin, and laminin, which implies the role of GZB in extracellular matrix (ECM) remodeling [8]. Therefore, the mechanisms by which GZB may contribute to plaque instability may include ECM degradation and/or induction of macrophage or smooth muscle cells apoptosis in the fibrous cap [9].

Interleukin 18 (IL-18), previously known as interferon-gamma (IFN-*γ*) inducing factor, is a proinflammatory member of IL-1 superfamily which plays a role in the initiation and progression of atherosclerosis [10]. Both clinical and experimental studies have supported its role in atherosclerotic plaque progression and destabilization [11, 12]. Nevertheless, it was described as an independent predictor of future adverse events in patients with ACS [13].

Another contributing factor to the formation of atherosclerotic plaques and may participate in their destabilization is chemokines [14]. In this regard, fractalkine (FKN) or CX3CL1 is a unique dual function chemokine that exists in two forms; a soluble form which acts as a chemoattractant and a membrane bound form acting as an adhesion molecule [15]. FKN was recently identified as an independent key factor in the pathogenesis of plaque vulnerability and subsequent plaque rupture [16]. 

 Hence, the current study was designed to determine the circulating levels of GZB in patients with ACS being compared with healthy control subjects. Furthermore, the associations between GZB with markers of inflammation and plaque destabilization (hsCRP, IL-18, and FKN) as well as other metabolic, anthropometric, and risk factors are being evaluated. 

## 2. Subjects and Methods

### 2.1. Subjects

 A total of 48 subjects (36 men and 12 postmenopausal women) were enrolled in the study, of which 36 nondiabetic ACS patients, being compared to 12 age- and sex-matched apparently healthy subjects as the control group. Twenty-two patients (61%) were diagnosed with ST segment elevation myocardial infarction (STEMI) and 14 (39%) with non ST segment elevation myocardial infarction (NSTEMI) or unstable angina (UA). STEMI, NSTEMI, and UA were diagnosed according to criteria stated by the consensus document of the Joint European Society of Cardiology/American College of Cardiology Committee for the redefinition of myocardial infarction [17]. Baseline characteristics of ACS patients and controls are given in Table 1. Patients were recruited from the Intensive Care Unit, Cardiology Department, El-Demerdash Hospital, Ain Shams University Educational Hospitals, Cairo, Egypt.

Subjects with fasting plasma glucose ≥7.0 mmol/L or with a previous history of diabetes mellitus, inflammatory diseases, autoimmune disorders, malignancy, hematological diseases, hepatic or renal diseases, acute or chronic infections, or administration of immunosuppressive drugs were excluded from this study.

 The study was carried out in accordance with the regulations and recommendations of the Declaration of Helsinki, being approved by the Committee on Medical Ethics of El-Demerdash hospital as well as Faculty of Pharmacy, Ain Shams University Ethical committee and informed consent was obtained from all participants. 

### 2.2. Methods

#### 2.2.1. Data Collection

A detailed family and medical history and drug treatment(s) were collected for all subjects. Systolic blood pressure (SBP), diastolic blood pressure (DBP), heart rate (HR), left ventricular ejection fraction (LVEF), and the incidence of prior ACS were obtained from medical records. Routine serum samples for creatine kinase (CK) and CK-MB isoenzyme were obtained at admission and at 6- to 8-hour intervals. Peak CK and peak CK-MB were recorded (Table 2). Hypertension (HTN) was defined as SBP > 140 mm Hg and/or DBP > 90 mm Hg and/or initiation of antihypertensive medication.

Demographic data and biochemical parameters of STEMI and NSTEMI/UA groups are demonstrated in Table 2. All patients received aspirin, clopidogrel, and intravenous heparin. Moreover, beta blockers, calcium channel blockers, angiotensin converting enzyme inhibitors, diuretics, nitrates, and statins were described according to the current guidelines. 

#### 2.2.2. Sample Preparation, Collection, and Storage

Peripheral blood samples (5 mL) were taken from ACS patients on day 1 (day of onset) and day 3 after onset. Samples were divided into 2 aliquots; the first part was collected on plain vacutainer tubes for serum preparation used for lipids profile, hsCRP, and IL-18 assay. The second aliquot was collected on EDTA for GZB and FKN assay. Both plasma and serum were divided into several aliquots, being stored at −80°C for subsequent assay.

#### 2.2.3. Laboratory Assessments

Lipids profile was measured by enzymatic method according to kits provided by Hannover, Germany. Serum hsCRP and IL-18 were quantified by enzyme linked immunosorbent assay (ELISA) technique using commercial available kit (hsCRP: Accubind, Momobind Inc., USA; IL-18: Wuhan Eiaab Science Co., China). Plasma GZB was assayed using Wkea Med Supplies Corp., NY, USA ELISA kit and plasma FKN ELISA kit was supplied by R&D Systems, Inc, Minneapolis, MN, USA. All ELISA procedures were done by Hyprep automated ELISA system (Hyperion Inc, Miami, FL) according to the manufacturer's instructions.

### 2.3. Statistical Analysis

The IBM statistical package for social sciences (SPSS) statistics (V.19.0, IBM Corp., USA, 2010) was used for data analysis. Continuous variables were presented as mean ± SEM and categorical ones as actual numbers and percentages. Comparisons between categorical variables were performed using chi-square test. Comparisons between two independent normally distributed mean groups were done using independent-samples *t*-test. Skewed data were analyzed by Mann-Whiteny *U* test. Changes in the serum or plasma levels of the inflammatory markers were evaluated with paired *t*-test or Wilcoxon matched pairs test. Correlations between markers were ascertained with Ranked Spearman test. Multiple stepwise regression analysis was done to identify risk factors that contribute to plasma GZB levels.

## 3. Results

### 3.1. Basic Characteristics of the Studied Groups

Serum levels of hsCRP, GZB, IL-18, and FKN showed significant elevation in ACS group when compared to the healthy control group at *P* < 0.001 (Table 1). Stepwise in the NSTEMI/UA and STEMI groups, hsCRP and GZB were significantly increased in the later group in comparison to the former (Table 2).

### 3.2. Changes in hsCRP, GZB, IL-18, and FKN Levels in ACS Groups

 Comparisons between hsCRP, GZB, IL-18, and FKN levels on day 1 and day 3 being compared to the control group levels are given in Table 3. Both STEMI and NSTEMI/UA groups showed the same dynamic changes in the measured biomarkers but with different degrees. In comparison to day 1, hsCRP levels significantly decreased on day 3 in either NSTEMI/UA or STEMI groups to 74% and 65%, respectively. Moreover, plasma GZB levels significantly increased to 118% in NSTEMI/UA and to 126% in the STEMI group in day 3 compared to day 1 in both groups, respectively. Serum IL-18 levels remained more or less constant in both groups. However, plasma FKN decreased significantly on day 3 by 8% in NSTEMI/UA and by 11% in STEMI group in comparison to its level in day 1.

### 3.3. Correlations between IL-18, GZB, and FKN Among Each Other and with Clinical Parameters of ACS Patients

**Figure 1:**
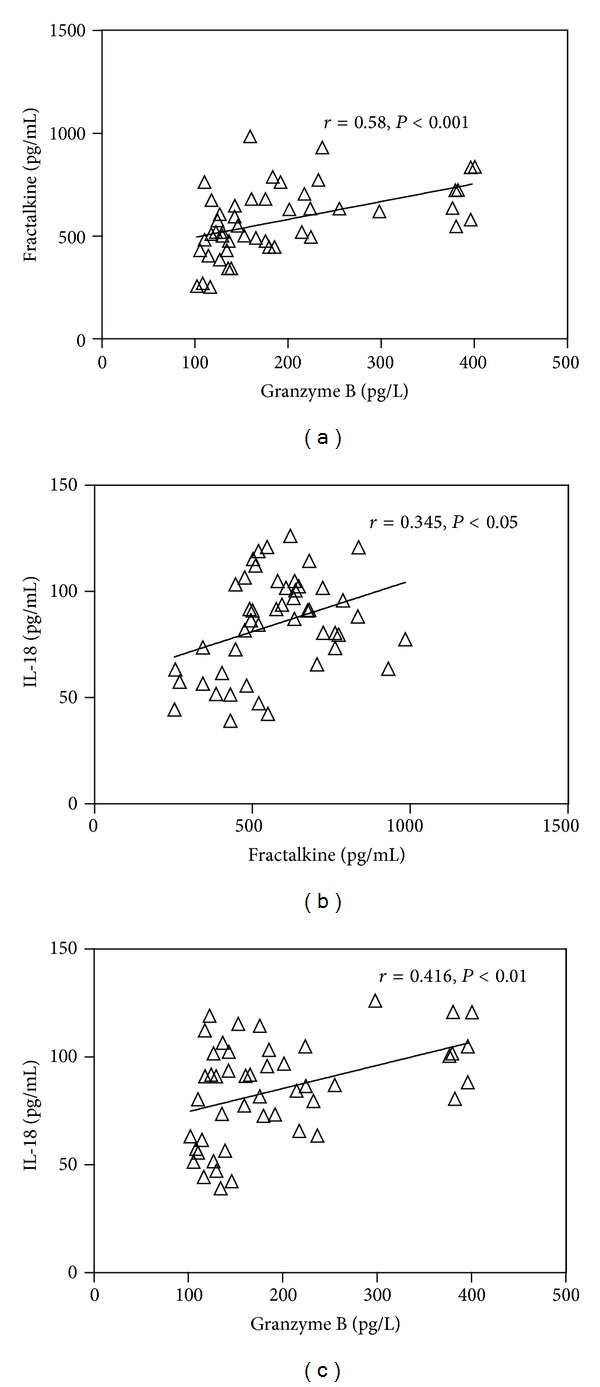
(a) Correlation between fractalkine (pg/mL) and granzyme B (pg/L), (b) correlation between fractalkine (pg/mL) and IL-18 (pg/mL), and (c) correlation between granzyme B (pg/L) and IL-18 (pg/mL).

As shown in Figure 1, a significant positive correlation is observed between GZB and FKN (*r* = 0.58, *P* < 0.001). Furthermore, IL-18 levels displayed a significant positive correlation with FKN (*r* = 0.345, *P* < 0.05) and GZB (*r* = 0.416, *P* < 0.01).

For the clinical parameters of ACS patients, as depicted in Table 4, correlation analyses also revealed significant correlations between GZB and SBP, DBP, peak CK, and peak CK-MB. However, serum IL-18 did not show significant correlation with any of anthropometric or clinical parameters in the ACS patient group. On the other hand, FKN levels showed significant positive correlations with BMI and peak CK-MB only.

### 3.4. Effect of Cardiovascular Risk Factors on Plasma GZB Levels in ACS Patients

 Next, we conducted multivariate regression analysis to examine the cardiovascular risk factors contributing to plasma granzyme B levels. We set plasma granzyme B levels as a dependent variable and age, gender, BMI, HTN, hypercholesterolemia, current smoker, and patient history of previous ACS as independent variables. As depicted in Table 5, HTN and patient history were independent risk factors that significantly affect plasma granzyme B levels in patients with ACS.

## 4. Discussion

Increased systemic inflammatory mediators in patients with stable atherosclerotic plaques are a major driving force for plaque disruption and hence the incidence of ACS syndrome [18]. However, other inflammatory conditions such as diabetes mellitus may affect circulating levels of those biomarkers [19, 20]. Therefore, the need for assessment of the inflammatory state in patients with ACS in absence of such interfering condition has been rising.

Since extracellular GZB was discovered, several *in vitro* studies have been done to verify its mechanisms of action but only a limited number of clinical studies evaluated its role as a circulating biomarker for cardiovascular diseases [21–23]. These studies attributed the rise in GZB levels to its nature as proapoptotic protein not as a novel proinflammatory marker.

 Accordingly, our study evaluated the association between the blood levels of known inflammatory cytokines like hsCRP, IL-18, and FKN with GZB in nondiabetic patients with acute cardiac events. We also focused on the temporal changes in levels of those biomarkers in patients with STEMI versus NSTEMI/UA to highlight the differential inflammatory response in different types of ACS and moreover to explain the impact of sample timing (day 1 and 3) on the recorded results.

The present study demonstrated a significant increase in blood levels of hsCRP, GZB, IL-18, and FKN in ACS patients compared to the control group. Elevated levels of GZB were detected in the plasma of patients with atherosclerosis, with the highest levels detected in patients with unstable plaques, lending support to the hypothesis that granzyme B influences plaque instability [23]. In addition, the consequences of elevated granzyme B levels may extend beyond plaque rupture. Satio et al. had described the novel role of elevated levels of GZB at day 14 after acute myocardial infarction (AMI) in postinfarct ventricular remodeling [24]. Moreover, the authors found that GZB protein expression was increased at the infarcted myocardium from day 3 to 17 after AMI.

Within the atherosclerotic plaques, GZB expression is not limited to CTLs and NK cells. Macrophages, foam cells, and smooth muscle cells (SMCs) also express GZB as well as it also can be detected extracellularly [9, 25]. Interestingly, GZB expression in the atherosclerotic plaque was found to be increased with disease severity [26]. Recently, Hendel et al. also identified that the reduced expression of the GZB endogenous inhibitor, proteinase inhibitor 9, in vascular SMCs is associated with the disease progression and that may increase SMCs susceptibility to GZB-induced apoptosis within the plaque [27]. Therefore, we found that GZB levels were increased significantly in STEMI group in comparison to NSTEMI/UA group.

Although Kondo and coworkers have proved that plasma GZB is elevated in patients with AMI [22], however, Tsuru et al. could not evaluate changes in plasma GZB concentration in UA patients where only few patients showed detectable results [28]. This may also support the significant difference in our plasma GZB levels between STEMI and NSTEMI/UA which probably may be due to proapoptotic nature of GZB associated with the significantly higher degree of cardiac necrosis, reflected by significant peak CK and peak CK-MB, and inflammation (hsCRP) observed in STEMI compared to NSTEMI/UA group.

 There is no expression of FKN in the normal coronary artery; however, FKN and its receptor CX3CR1 are highly expressed in atherosclerotic lesions [29]. As was the case in our study, Li et al. found higher FKN levels in patients with ACS when compared to control subjects or even when compared to patients with stable angina [16].

 Ziakas et al. identified higher peak values of acute phase reactants in patients with STEMI versus NSTEMI/UA [30]. For hsCRP, previous studies demonstrated significantly higher admission and peak values in subjects with STEMI compared to NSTEMI and in AMI compared to UA [31, 32]. This comes in line with our finding where hsCRP levels were significantly higher in STEMI patients compared to NSTEMI/UA patients. For IL-18, our finding was confirmed by Brunetti et al. who reported that serum levels of IL-18 are not affected by ACS diagnosis [33]. Furthermore, a recent study revealed that the circulating levels of FKN were not statistically different between AMI and UA groups [16].

GZB release after ACS showed a time dependent kinetics. An *in vitro *study identified that the production of GZB from cultured peripheral blood mononuclear cells is time dependent, where GZB levels in culture media gradually increased up to 48 hours after incubation [28]. Moreover, GZB showed peak plasma levels on day 7 after AMI [22]. This comes in agreement with our results, where plasma GZB levels significantly increased on day 3 compared to day 1 in both STEMI and NSTEMI/UA groups.

In the study conducted by Ghanavatian et al. hsCRP levels decreased 12 hours after admission in patients with NSTEMI [34]. In our study hsCRP decreased after 3 days in comparison to day 1 in both groups. To the best of our knowledge, this is the first study to compare plasma FKN levels in timed samples after ACS. On the other hand, a study of inflammatory cytokines imbalance in patients with ACS revealed that IL-18 levels were significantly high on admission and remained unchanged after 12 and 24 hours [33]. This was the case in our study where serum IL-18 did not change significantly from day 1 to day 3 in both groups.

Correlation analyses showed significant positive correlation between FKN levels and GZB. This association could be explained in context of the results of Kyaw and coworkers who concluded that CD8+ T lymphocytes, the most abundant inflammatory cells in advanced atherosclerotic lesions, promote plaque vulnerability via GZB-mediated apoptosis of macrophages, SMCs, and endothelial cells [35]. Previously, it was shown that the interaction between FKN and its receptor is the main player in trafficking such GZB producing CD8+ T lymphocytes into the vascular lesions [36] that suggests a strong relationship between FKN and GZB levels as was demonstrated in our results.

The extracellular role of GZB indicates that GZB may have an indirect mechanism for eliciting cytokine release. Recently, it was demonstrated that granzyme B processes IL-1*α* into a significantly more potent proinflammatory fragment [37]. Additionally, a direct relationship between IL-18 and GZB was identified by Omoto et al. who found that GZB as a serine protease cleaves inactive pro-IL-18 at the same residue cleaved by its documented activator caspase-1; hence GZB could be considered as a novel activator of the proinflammatory IL-18 [38]. As a result, accumulation of the extracellular GZB may result in elevation of serum IL-18 levels which is consistent with our results.

To the best of our knowledge, no direct relationship was reported between FKN and IL-18. However, previous studies showed that IFN-*γ* stimulates FKN expression in vascular endothelial cells [39, 40]. Moreover, other members of IL-1 superfamily such as IL-1*β* were also involved in FKN induction [41]. These relationships may illustrate the positive significant correlation between FKN and IL-18 in our study.

## 5. Conclusions

There might be some sort of interplay between GZB, IL-18, and FKN in the pathogenesis of ACS. The current study also confirmed the different inflammatory response in STEMI patients with respect to NSTEMI/UA patients. Indeed, further large scale prospective studies are required to demonstrate the possible use of plasma GZB in risk stratification after myocardial ischemia.

## Figures and Tables

**Table 1:** Demographic data and clinicopathological parameters of the studied groups.

Groups	Controls	ACS	*P*
Factor/*n*	12	36	
Age (years)	47.92 ± 1.42	50.4 ± 0.5	NS
Sex (M/F)	10/2	26/10	NS
BMI (kg/m^2^)	28 ± 0.7	28.4 ± 0.3	NS
TAG (mmol/L)	0.96 ± 0.05	1.26 ± 0.02	<0.01
TC (mmol/L)	4.4 ± 0.14	5.23 ± 0.08	<0.001
HDL-C (mmol/L)	1.11 ± 0.04	0.92 ± 0.02	<0.01
LDL-C (mmol/L)	2.82 ± 0.09	3.65 ± 0.06	<0.001
LDL-C/HDL-C	2.6 ± 0.1	4.1 ± 0.1	<0.001
TC/HDL-C	4 ± 0.1	5.8 ± 0.2	<0.001
hsCRP (mg/L)	0.7 ± 0.1	9.4 ± 0.6	<0.001
GZB (pg/L)	122.3 ± 4.2	193.4 ± 13.3	<0.001
IL-18 (pg/mL)	53.7 ± 2.8	94.5 ± 3.7	<0.001
FKN (pg/mL)	388.8 ± 28.8	637.2 ± 22.7	<0.001

Results are mean ± SEM.

HDL-C: high density lipoprotein cholesterol; LDL-C: low density lipoprotein cholesterol; NS: not significant; TAG: triacylglycerol; TC: total cholesterol.

**Table 2:** Demographic data and biochemical parameters in STEMI and NSTEMI/UA groups.

Groups	NSTEMI/UA	STEMI	*P*
Factor/*n*	14	22	
Age (years)	50.5 ± 0.75	50.32 ± 0.63	NS
Sex (M/F)	8/6	18/4	NS
BMI (kg/m^2^)	29 ± 0.68	27.95 ± 0.31	NS
Current smoker, *n* (%)	5 (36% )	9 (41%)	NS
Prior ACS, *n* (%)	11 (78%)	5 (23%)	*P* < 0.01
SBP (mmHg)	130.71 ± 6.06	125 ± 5.29	NS
DBP (mmHg)	80.7 ± 5.6	76.8 ± 2.7	NS
Hypertension, *n* (%)	8 (57%)	12 (55%)	NS
HR (bpm)	83.3 ± 2.1	85.3 ± 3.2	NS
LVEF (%)	51.3 ± 3.1	47.6 ± 2.2	NS
Peak CK (IU/L)	407.8 ± 121.2	1874.4 ± 231	<0.001
Peak CK-MB (IU/L)	88.3 ± 22.6	235.6 ± 24.3	<0.001
Treatment (BB/CCB/ACEI/diuretics/nitrates/statins)	13/0/8/1/4/13	11/2/13/4/2/19	
TAG (mmol/L)	1.23 ± 0.04	1.27 ± 0.02	NS
TC (mmol/L)	5.04 ± 0.13	5.34 ± 0.09	NS
HDL-C (mmol/L)	0.9 ± 0.04	0.92 ± 0.03	NS
LDL-C (mmol/L)	3.52 ± 0.12	3.73 ± 0.05	NS
LDL-C/HDL-C	4 ± 0.2	4.15 ± 0.2	NS
TC/HDL-C	5.7 ± 0.3	5.9 ± 0.2	NS
Hypercholesterolemia, *n* (%)	5 (36%)	15 (68%)	NS
hsCRP (mg/L)	6.4 ± 0.5	11.3 ± 0.6	<0.001
GZB (pg/L)	158.1 ± 9.7	254.6 ± 21.8	<0.01
IL-18 (pg/mL)	99.8 ± 3.6	92 ± 3.6	NS
FKN (pg/mL)	598.2 ± 32	662.1 ± 30.3	NS

Results are mean ± SEM.

ACEI: angiotensin converting enzyme inhibitor; BB: beta blocker; CCB: calcium channel blocker; CK: creatine kinase; CK-MB: creatine kinase MB fraction; DBP: diastolic blood pressure; HDL-C: high density lipoprotein cholesterol; HR: heart rate; LDL-C: low density lipoprotein cholesterol; LVEF: left ventricular ejection fraction; NS: not significant; SBP: systolic blood pressure; TAG: triacylglycerol; TC: total cholesterol.

**Table 3:** Changes in blood levels of measured markers in STEMI and NSTEMI/UA groups.

Groups	Controls	NSTEMI/UA		STEMI	
Factor/*n*	12	14		22	
		First day	Third day	First day	Third day
hsCRP (mg/L)	0.7 ± 0.1	6.4 ± 0.5*	4.7 ± 0.5^∗‡‡^	11.3 ± 0.6*	7.3 ± 0.6^∗¶^
GZB (pg/L)	122.3 ± 4.2	158.1 ± 9.7^†^	187.3 ± 10.7^∗‡^	254.6 ± 21.8*	320.5 ± 22.7^∗¶^
IL-18 (pg/mL)	53.7 ± 2.8	99.8 ± 3.6*	97.9 ± 4.7*	91.2 ± 5.6*	93.5 ± 5.2*
FKN (pg/mL)	388.8 ± 28.8	598.2 ± 32*	548.9 ± 26^∗§^	662.1 ± 30.3*	587 ± 25.1^∗¶^

Results are mean ± SEM.

*P < 0.001 ACS subgroup compared to control group.

^†^ *P* < 0.01 ACS subgroup compared to control group.

^‡‡^ *P* < 0.001 third day NSTEMI/UA compared to first day NSTEMI/UA.

^‡^ *P* < 0.01 third day NSTEMI/UA compared to first day NSTEMI/UA.

^§^ *P* < 0.05 third day NSTEMI/UA compared to first day NSTEMI/UA.

^¶^ *P* < 0.001 third day STEMI compared to first day STEMI.

**Table 4:** Correlation coefficients of GZB, IL-18, and FKN with anthropometric and clinical data in ACS patients.

Factor	Correlation coefficient (*r*)		
	GZB (pg/L)	IL-18 (pg/mL)	FKN (pg/mL)
Age (years)	−0.064^NS^	0.285^NS^	0.011^NS^
BMI (kg/m^2^)	−0.035^NS^	−0.211^NS^	0.622**
SBP (mmHg)	0.469**	0.087^NS^	0.206^NS^
DBP (mmHg)	0.416*	0.142^NS^	0.301^NS^
LVEF (%)	−0.076^NS^	0.015^NS^	−0.178^NS^
HR (bpm)	0.177^NS^	−0.128^NS^	0.121^NS^
Max. total CK (IU/L)	0.481**	−0.003^NS^	0.266^NS^
Max. CK-MB (IU/L)	0.404*	−0.156^NS^	0.402*
hsCRP (mg/L)	0.59**	0.564**	0.514**

*Significant at *P* < 0.05 level; **significant at *P* < 0.01 level; NS: nonsignificant correlation.

**Table 5:** Predicting factors for plasma GZB levels in ACS patients.

Risk factor	*β* coefficient	*P*
Age (years)	0.067	0.648
Gender	−0.079	0.591
BMI (kg/m^2^)	−0.149	0.305
HTN	0.437	0.004
Hypercholesterolemia	0.257	0.101
Current smoker	0.093	0.53
Patient history	−0.368	0.015

BMI: body mass index; HTN: hypertension.
